# Insights into the rapid metabolism of *Geobacillus* sp. LC300: unraveling metabolic requirements and optimal growth conditions

**DOI:** 10.1007/s00792-023-01319-x

**Published:** 2023-12-01

**Authors:** Emil Ljungqvist, Jeanett Daga-Quisbert, Antonius van Maris, Martin Gustavsson

**Affiliations:** 1 Department of Industrial Biotechnology, School of Engineering Sciences in Chemistry, Biotechnology and Health, KTH Royal Institute of Technology, Alba Nova University Center, 106 91 Stockholm, Sweden; 2 Center of Biotechnology, Faculty of Science and Technology, Universidad Mayor de San Simón, Cochabamba, Bolivia

**Keywords:** *Geobacillus*, Thermophile, Defined medium, Quantitative physiology, Metabolism, Bioprocess

## Abstract

This study investigated the metabolism of *Geobacillus* sp. LC300, a promising biorefinery host organism with high substrate utilization rates. A new defined medium was designed and tested that allows for exponential growth to elevated cell densities suitable for quantitative physiological studies. Screening of the metabolic requirements of *G.* sp. LC300 revealed prototrophy for all essential amino acids and most vitamins and only showed auxotrophy for vitamin B12 and biotin. The effect of temperature and pH on growth rate was investigated, adjusting the optimal growth temperature to several degrees lower than previously reported. Lastly, studies on carbon source utilization revealed a capability for fast growth on several common carbon sources, including monosaccharides, oligosaccharides, and polysaccharides, and the highest ever reported growth rate in defined medium on glucose (2.20 h^−1^) or glycerol (1.95 h^−1^). These findings provide a foundation for further exploration of *G.* sp. LC300's physiology and metabolic regulation, and its potential use in bioproduction processes.

**Supplementary Information:**

The online version contains supplementary material available at 10.1007/s00792-023-01319-x.

## Introduction

Thermophilic microorganisms possess several beneficial traits for microbial bioproduction processes. Their high growth temperature gives lowered risk of mesophilic contaminations, lowered bioreactor cooling costs, and facilitated purification of volatile products (Hussein et al. 2015). *Geobacillus* is a genus of thermophilic Gram-positive bacteria, with optimal growth temperatures between 44 and 75 °C (Hussein et al. 2015). Combining the bioprocessing advantages of thermophiles with their capacity to consume a wide range of substrates, including monosaccharides, polysaccharides, and fatty acids (De Maayer et al. 2014; Hussein et al. 2015), makes *Geobacilli* promising biorefinery hosts.

The use of complex media can, depending on the requirement for ingredients such as yeast extract or peptone, negative influence cost and reproducibility through lot-to-lot variation, which in turn can cause batch-to-batch productivity variations in bioproduction processes (Zhang and Greasham 1999). By composing a chemically defined growth medium, the composition of nutrients, trace elements, and other factors that influence microbial growth and behavior can be controlled. Such control is paramount when quantitatively studying microbial physiology, metabolism, and regulation. To date, only a few defined growth media have been determined for the *Geobacillus* genus (Rowe, Goldberg and Amelunxen, 1975; San Martin et al. 1992; Singleton et al. 2019), and in-depth physiological studies of the genus are consequently limited.

*Geobacillus* sp. LC300 is a recently discovered aerobic thermophile, displaying fast substrate utilization rates on monosaccharides that could be obtained from lignocellulosic feedstocks (Cordova et al. 2015). These high rates make *G.* sp. LC300 an interesting biorefinery host organism. However, the previously reported growth media used for the cultivation of *G.* sp. LC300 (Cordova et al. 2015, 2017; Cordova and Antoniewicz 2016; Ljungqvist and Gustavsson 2022) contains both yeast extract as well as a range of vitamins. Furthermore, the concentrations of the components in the medium are relatively low and the exact metabolic requirements of *G.* sp. LC300 are hitherto unknown, which prohibits carbon-limited growth at high cell densities and thereby quantitative physiological studies in, for instance, chemostat cultures.

The aim of this study was to design a new medium based on the *G.* sp. LC300 metabolic requirements predicted by the genome-scale model *iGEL604* (Ljungqvist and Gustavsson 2022). Subsequently, the vitamin requirements of *G.* sp. LC300 were screened. The effect on growth rate of temperature and pH was investigated using a design of experiments approach. Lastly, growth of *G.* sp. LC300 was investigated on a range of carbon sources, including monosaccharides, oligosaccharides, polysaccharides, and organic acids.

## Materials and methods

### Growth media

Wolfe’s medium (Swarup et al. 2014), previously used for the propagation of *G.* sp. LC300, (Cordova et al. 2015, 2017; Cordova and Antoniewicz 2016; Ljungqvist and Gustavsson 2022) was used as a basis for medium development in the present work. This consisted of (per liter): 0.5 g K_2_HPO_4_, 0.3 g KH_2_PO_4_, 0.5 g NH_4_Cl, 0.5 g NaCl, 0.05 g yeast extract (Difco laboratories Art. No: 212720), 40 mL 1 M Tris–HCl (pH 8). After autoclaving, the following chemicals were added by sterile filtration: 0.24 g MgSO_4_ × 7H_2_O, 0.033 g CaCl_2_ × 2H_2_O, 5 mL Wolfe’s minerals solution, 5 mL Wolfe’s vitamins solution, and 10 g D-glucose. Wolfe’s minerals contained (per liter): 0.5 g EDTA, 3.0 g MgSO_4_ × 7H_2_O, 0.5 g MnSO_4_ × H_2_O, 1.0 g NaCl, 0.1 g FeSO_4_ × 7H_2_O, 0.1 g Co(NO_3_)_2_ × 6H_2_O, 0.1 g CaCl_2_ × 2H_2_O, 0.1 g ZnSO_4_ × 7H_2_O, 0.01 g CuSO_4_ × 5H_2_O, 0.01 g AlK(SO_4_)_2_, 0.01 H_3_BO_3_, 0.01 g Na_2_MoO_4_ × 2H_2_O, 0.001 g Na_2_SeO_3_, 0.01 g Na_2_WO_4_ × 2H_2_O, 0.022 g NiSO_4_ × 6H_2_O. Wolfe’s vitamins contained (per liter): 2.0 mg Folic acid, 10 mg pyridoxine hydrochloride, 5.0 mg riboflavin, 2.0 mg biotin, 5.0 thiamine hydrochloride, 5.0 mg nicotinic acid, 5.0 mg calcium pantothenate, 0.1 mg vitamin B12, 5.0 mg p-aminobenzoic acid, 5.0 mg thioctic acid, 900 mg KH_2_PO_4_. During this study, the Wolfe’s medium was iteratively modified as described for individual experiments in the results section. The final composition of the designed minimal LC300 growth medium (MLGM) can be found in Table 2.

### Shake flask cultivations

Seed cultivations were prepared by inoculating glycerol working stocks kept at − 80 °C to 25 mL of the relevant culture medium for each individual experiment, supplemented with 1 g L^−1^ yeast extract. The cultures were then incubated in 250 mL baffled Erlenmeyer flasks placed in an orbital shaker (ES-80, GrantBio, Shepreth, UK) with the air temperature set to 68 °C and 200 rpm. When an optical density at 600 nm (OD_600_) of 1 was reached, cells were transferred into medium without yeast extract to an OD_600_ of approximately 0.03, and incubated again (68 °C, 200 rpm) to an approximate OD_600_ of 1. Finally, these cultures were used to inoculate new 250 mL baffled Erlenmeyer flasks containing 25 mL of culture medium with the appropriate composition to an OD_600_ of approximately 0.03, and the growth phenotype was monitored.

### Vitamin essentiality screening

To screen for vitamin essentiality, shake flask cultivations were performed as described above, with the following modification. After preculture harvest, cells were centrifuged (Z206A, Hermle, Gosheim, Germany) for 5 min at 3000*g* at room temperature and washed twice with culture medium without vitamins. These washed cells were then used to inoculate flasks, each containing medium with one of the ten Wolfe’s vitamins excluded. For each medium composition, growth was assessed by OD_600_ measurements starting from approximately 0.03 at the time of inoculation, and the maximum biomass specific growth rate was calculated from the exponential growth phase.

### Bioreactor cultivations

Seed cultivations in 25 ml medium were performed as described in “Shake flask cultivations” above. When an OD_600_ of around 1 was reached, cultures were transferred into 1 L baffled Erlenmeyer flasks containing 100 mL of cultivation medium to a starting OD_600_ of 0.03. These flasks were incubated until an approximate OD_600_ of 0.1 was reached and then placed at 4 °C overnight. The next morning, the flasks were transferred back to the incubator (68 °C, 200 rpm) and cultures were grown until an approximate OD_600_ of 1 was reached. These exponentially growing cultures were used to inoculate six parallel 1.5 L stainless steel stirred-tank bioreactors (Greta, Belach Bioteknik AB, Skogås, Sweden) containing 750 mL cultivation medium to a starting OD_600_ of approximately 0.03, as previously described (Ljungqvist and Gustavsson 2022). The temperature of the growth medium was regulated at set-points as indicated (± 0.1 °C) through automatic recirculation of warm water (85 °C) in the bioreactor jackets. The starting pH was set using 4 M H_2_SO_4_ and then regulated by automatic titration using 4 M NaOH. The dissolved oxygen tension was kept above 30% saturation by automatically increasing stirrer speed (200–1500 rpm) and step-wise increase of aeration (0.2–2 L min^−1^). Foaming was reduced by manual addition of anti-foam (Breox B125, BASF) when required.

### Analyses

To monitor biomass formation, samples were collected from shake flask (1 mL) and bioreactor (2 mL) cultivation every 20 min. In bioreactor cultivations, the same samples were also used to monitor substrate consumption and metabolite excretion. Biomass was monitored through OD_600_ measurements in a spectrophotometer (Genesys 20, Thermo Scientific) after diluting to OD_600_ 0.06 – 0.25 in saline solution (0.9% w/v NaCl). In bioreactor cultivations, cell dry weight (CDW) analysis was performed in triplicates at 4 time-points throughout the cultivations by centrifugation of 10 mL culture sample at 4500 rpm (Z206A, Hermle, Gosheim, Germany), washing of the cell pellet in demineralized water, followed by overnight drying at 110 °C. The OD_600_:CDW conversion ratio was determined to 2.7, which was then used to convert remaining OD_600_ values to CDW. To determine substrate and metabolite concentrations, samples were centrifuged at 13,000 rpm for 5 min in a tabletop microcentrifuge (Biofuge A, Heareus Spatech), transferred to new microcentrifuge tubes and then centrifuged again. The resulting supernatants were analyzed by HPLC (Waters, Milford, MA, USA) with an Aminex HPX-87H column (Bio-Rad, Hercules, CA, USA). Separation was performed at 60 °C with 5 mM H_2_SO_4_ as a mobile phase at a flow rate of 0.6 mL min^−1^. Sugars were quantified using a refractive index detector (Waters) at 410 nm, and organic acids were quantified through absorbance at 210 nm using a diode array detector (Waters).

### Temperature and pH optimization

A design of experiments approach was used for evaluation of cultivation parameter effects on growth rate. Two factors were investigated, temperature and pH of the cultivation medium. MODDE Pro 13.0 (Sartorius, Germany) was used to plan and evaluate the study. A circumscribed central composite (CCC) design with three center points was chosen, totaling 11 experiments. This allowed modeling of both interactions between the studied factors and their individual quadratic effects (Eq. 1). Partial least squares regression was performed to fit the measured response to the model. The included factors in the final model (Eq. 2) were selected based on maximizing the goodness of prediction (*Q*^2^) while keeping the difference between goodness of fit (*R*^2^) and *Q*^2^ below 0.2 to avoid overfitting (Eriksson et al. 2000). Factors in Eqs. 1 and 2 are linearly scaled so that CCC corner points (Temp 62 °C, 68 °C; pH 6.5, 7) correspond to − 1 and 1 for each factor, with the center point 65 °C, pH 6.75 corresponding to 0 for both factors. Statistical evaluation (ANOVA) of the model fit can be seen in Supplementary Table 1.

1 $$\mu =A+B\times \mathrm{Temp}+C\times \mathrm{pH}+D\times {\mathrm{Temp}}^{2}+E\times {\mathrm{pH}}^{2}\times \mathrm{Temp}\times \mathrm{pH},$$

2 $$\mu =2.127-0.203\times \mathrm{Temp}+0.008\times \mathrm{pH}-0.180\times {\mathrm{Temp}}^{2}-0.067\times {\mathrm{pH}}^{2}.$$

## Results and discussion

### Design of a defined growth medium

The macro-elemental composition previously used for genome-scale model *iGEL604* (Ljungqvist and Gustavsson 2022) was used as a baseline to evaluate the metabolic requirements of *G*. sp. LC300 (supplementary Table 2). Based on this predicted elemental composition, concentrations of all medium components excluding the carbon source were selected to support 5 g L^−1^ of biomass with a theoretical excess of at least 25%. This was done to ensure that the medium would support cultivations of *G. sp. LC300* up to 5 g L^−1^ with the carbon source being the only limiting nutrient. For medium components that are not directly coupled to the macro-elemental composition (i.e., vitamins and trace metals), defined media for *Escherichia coli* (M9)*, Saccharomyces cerevisiae*, and *Parageobacillus thermoglucosidasius* (Singleton et al. 2019) were studied to determine suitable starting concentrations. Based on this comparison, it was found that the Wolfe’s medium was relatively lean in these components, which could lead to limitations at higher cell densities. Consequently, the Wolfe’s medium was modified by doubling the original concentrations of magnesium, calcium, and Wolfe’s mineral solution, while the addition of Wolfe’s vitamins was increased tenfold. As is pointed out by Egli (2015), media designed for growth of aerobic microorganisms commonly severely lacks a sufficient iron supply. Thus, the iron content of the Wolfe’s medium was increased 20-fold to better support the rapid aerobic metabolism of *G.* sp. LC300. Furthermore, to make the medium chemically defined, the small amount of yeast extract added to Wolfe’s medium was excluded. Finally, the original Wolfe’s medium relies on Tris buffer to achieve sufficient buffering capacity in flask experiments. However, Tris buffer has been shown to inhibit reactions involved in protein biosynthesis in vitro (Good et al. 1966), and could have detrimental effects on cell physiology when used in growth media. When this was tested by changing buffer in the Wolfe´s medium from Tris–HCl to 50 mM MOPS while maintaining the initial pH at 6.9, a 15% increase in maximum growth rate on glucose was observed (data not shown). Consequently, MOPS was chosen as buffer for the new medium.

**Fig. 1:**
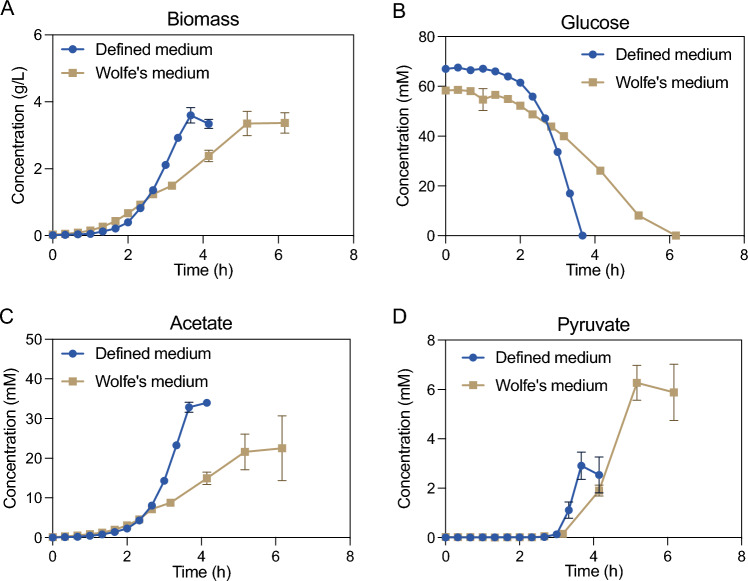
Growth, substrate consumption, and byproduct formation of *G.* sp. LC300 cultivated in bioreactors on two growth media (defined medium supplemented with Wolfe’s vitamins, and Wolfe’s medium), y-axes represent concentrations of biomass (**A**), glucose (**B**), acetate (**C**), and pyruvate (**D**), and *x*-axes represents time from inoculation. Data points show mean of duplicate bioreactor cultivations performed at 68 °C, pH 7 with error bars representing mean deviation

To evaluate the defined medium, bioreactor cultivations at 68 °C using 10 g L^−1^ glucose were compared to corresponding cultivations using Wolfe’s medium (Fig. 1). When cultivated in Wolfe’s medium, the cells displayed exponential growth with a growth rate of 1.56 (± 0.10) h^−1^ to 1 g_DW_ L^−1^, followed by linear growth to approximately 3.2 g_DW_ L^−1^. In contrast, in the defined medium, exponential growth was sustained with a growth rate of 1.70 (± 0.09) h^−1^ until 2 g_DW_ L^−1^, at which point oxygen transfer became limiting resulting in linear growth to 3.5 g_DW_ L^−1^. Similarly to the growth rate, the biomass specific glucose consumption rate in the exponential phase was higher in the defined medium (25.5 ± 1.26 mmol g_DW_^−1^ h^−1^) than in Wolfe’s medium (16.1 ± 0.70 mmol g_DW_^−1^ h^−1^). In both media, the sole byproduct was acetate, with biomass specific acetate production rates of 9.94 (± 0.97) mmol g_DW_^−1^ h^−1^ in the defined medium and 7.11 (± 0.84) mmol g_DW_^−1^ h^−1^ in Wolfe’s medium. In Wolfe’s medium, the exponential phase ends when 1 g_DW_ L^−1^ is reached, at which point the nitrogen is depleted (see supplementary materials for calculations). Following the nitrogen depletion, additional byproducts apart from acetate are produced, such as pyruvate (Fig. 1D) and small amounts of lactate (not shown). The same byproduct production pattern was observed in the defined medium upon oxygen limitation, i.e., after 3 h in Fig. 4C, D, with a sharp increase in pyruvate excretion. The results show that *G.* sp. LC300 can sustain fast growth to elevated cell densities without yeast extract supplementation. Consequently, when provided with necessary vitamins, functional metabolic pathways exist for the synthesis of all other biomass precursors.

### Elucidating vitamin requirements of *G.* sp. LC300

**Fig. 2:**
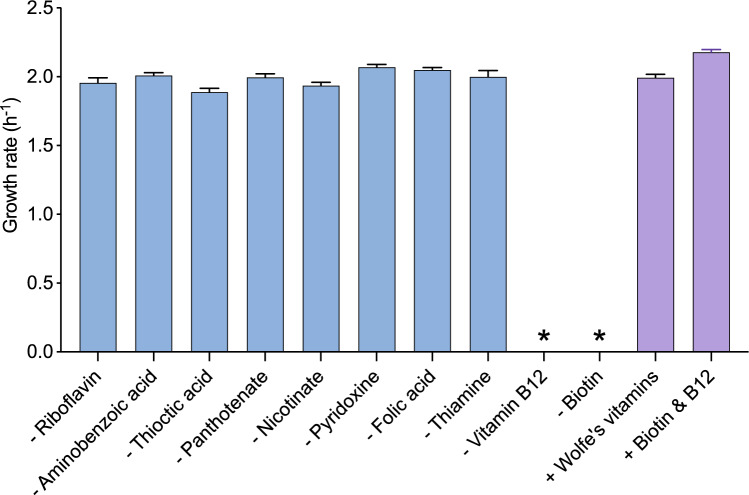
Achieved growth rates when excluding individual vitamins from the Wolfe’s vitamin mix (blue bars) or supplementing only specified vitamins (purple bars). Bars show the mean of triplicate shake flask cultivations, with error bars indicating standard deviation. *Exponential growth observed only for < 3 generations

With robust growth in a chemically defined growth medium, studies of the vitamin requirements of *G*. sp. LC300 could be performed, which was not possible in the previously used *G.* sp. LC300 complex growth medium (Swarup et al. 2014). To evaluate *G.* sp. LC300 vitamin prototrophy, cells were grown in ten modified medium compositions, each excluding a single vitamin from the Wolfe’s vitamin mix. The impact from the removal of each vitamin on the growth rate was observed (Fig. 2). When excluding either biotin or vitamin B12, exponential growth was only observed for approximately 3 generations, followed by linear growth. This growth pattern suggests an inability to synthesize these vitamins with remaining (non-exponential) growth due to intracellular dilution of the vitamins carried over from inoculation as the cells divide. The observed vitamin B12 auxotrophy confirms the earlier predictions of *iGEL604* (Ljungqvist and Gustavsson 2022), suggesting an incomplete vitamin B12 biosynthesis pathway in *G.* sp. LC300. Removing any of the other Wolfe’s vitamins had no significant effect on the growth rate, suggesting that *G.* sp. LC300 can readily synthesize these vitamins or that they are not essential for growth under the studied conditions. When adding only vitamin B12 and biotin to the growth medium, the growth pattern of the medium with all vitamins added was recovered (Fig. 2). Thus, the results indicate that *G.* sp. LC300 is auxotrophic for both biotin and vitamin B12, and a minimal medium should include both these vitamins.

**Fig. 3:**
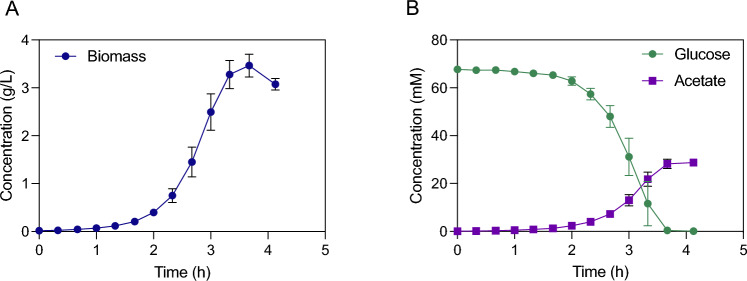
Growth, glucose consumption, and acetate accumulation of *G.* sp. LC300 cultivated in defined medium supplemented with biotin and vitamin B12. *Y*-axes represent concentrations of biomass (**A**) and metabolites glucose and acetate (**B**). *X*-axes represent time after inoculation. Data points show mean of quadruplicate bioreactor cultivations, with error bars representing standard deviation. Cultivations were performed at 68 °C, pH 7

To confirm that biotin and vitamin B12 are the only required vitamins and to investigate the impact of this medium change on metabolism, a bioreactor cultivation was performed. Similar to bioreactor cultivations in defined medium supplemented with all Wolfe’s vitamins (Fig. 1), supplementation with only biotin and vitamin B12 resulted in a maximum biomass specific growth rate of 1.76 (± 0.05) h^−1^ (Fig. 3). Exponential growth was sustained to a cell concentration of 2 g L^−1^, when oxygen transfer again became limiting. Acetate was the sole byproduct during the exponential phase, with a specific acetate production rate of 8.9 (± 0.2) mmol g_DW_^−1^ h^−1^, and glucose was consumed with a specific rate of 25.8 (± 1.1) mmol g_DW_^−1^ h^−1^. This shows that supplementation with only biotin and vitamin B12 is sufficient to achieve the same growth phenotype and biomass yield as cultures supplemented with the full Wolfe’s vitamin mix.

One notable difference was the 20% decrease in growth rate when using bioreactors compared to the shake flask screening experiments (1.76 h^−1^ versus 2.18 h^−1^). This discrepancy was hypothesized to be caused by differences in growth conditions between shake flasks and the better controlled environment in the bioreactors. When investigated, a significantly lower temperature was discovered in the shake flask cultivation medium (65 °C) compared to the incubator atmosphere (68 °C). This difference is likely the effect of evaporation drawing energy from the culture medium, reducing the temperature of the liquid compared to the incubator air temperature set point. These results seem to indicate that growth at 65 °C is faster than the previously reported optimum temperature of 68 °C. Furthermore, in shake flasks, growth ceased after reaching 2 g_DW_ L^−1^, likely a result of low pH due to acetate production. Since the effect of pH on growth has not previously been studied and the abovementioned results warrant a reinvestigation of the growth temperature, further investigation was required to determine optimal cultivation settings for *G.* sp. LC300.

### Effect of temperature and pH on growth rate

**Fig. 4:**
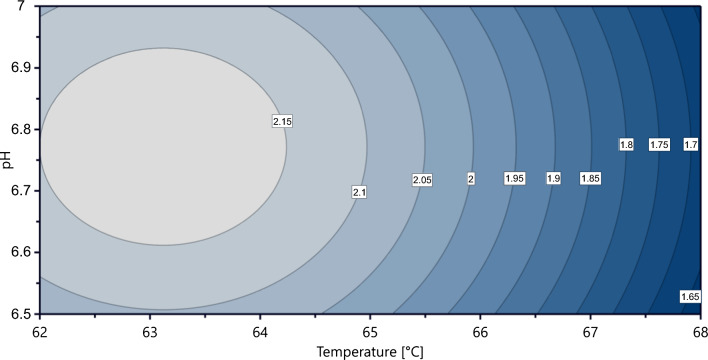
Design of experiments to determine optimal growth parameters. Modeled growth rate (h^−1^) as a function of temperature and pH

To investigate the optimal growth conditions for *G*. sp. LC300, a design of experiments approach was utilized. The circumscribed central composite design was employed to determine nine combinations of temperature and pH using the MODDE 13.0 software. Each temperature and pH combination was used for a cultivation, and the maximum growth rate in each cultivation was recorded. A model capable of predicting the growth rate based on temperature and pH was then constructed from the resulting data. Figure 4 presents the model's predicted growth rates, showing an optimal growth temperature plateau between 62 and 64 °C, with a maximum growth rate of 2.15 h^−1^. Notably, this growth rate in bioreactors matched the originally reported value for *G.* sp. LC300 by Cordova et al*.* at a reported temperature in shake flask cultivations of 72 °C (Cordova et al. 2015), highlighting the challenge in accurately reproducing thermophilic growth conditions between different labs and experimental setups. The optimal pH range is slightly below neutral, with the highest growth rate predicted between pH 6.6 and 6.9. The model does not predict a significant interaction between pH and temperature in the tested ranges. The model coefficients are shown in Eq. 2, and a statistical evaluation of the model predictions is shown in Supplementary Table 1.

**Fig. 5:**
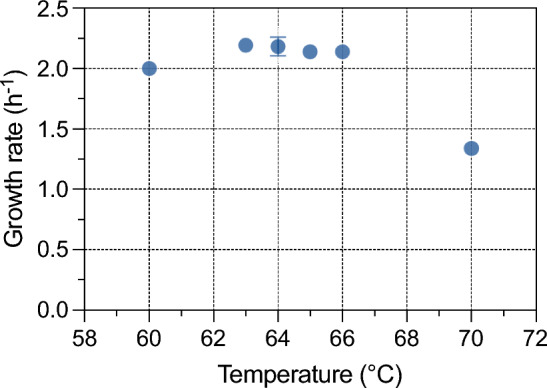
Measured maximum biomass specific growth rates at set temperatures with a constant pH of 6.75. Points show mean of duplicate bioreactor cultivations, with error bars displaying mean deviation. Extreme points (60 °C, 70 °C) show results from single bioreactor cultivations

The influence of temperature and pH on microbial growth rate has been widely studied (Davey 1994; Membré et al. 2005). While both factors commonly exhibit quadratic effects, a more pronounced decrease in growth rate is often observed at temperatures above the optimal than below (Huang et al. 2011). This asymmetrical relationship poses a challenge when using quadratic models, as is assumed by MODDE, resulting in inaccurate predictions of growth rates in temperatures around and above optimal. To address this issue, we conducted additional cultivations at the optimal pH of 6.75 and temperatures of 63 °C, 64 °C, and 66 °C to explore the predicted optimal growth temperature plateau at a higher resolution. The results of these cultivations (Fig. 5), show an extension of the plateau to 66 °C, and with a previously determined growth rate of 1.34 h^−1^ at 70 °C (Supplementary Table 3), and 1.76 h^−1^ at 68 °C and pH 7.0 (Fig. 3), these results suggest a large reduction of growth rate above 66 °C. To keep a > 1 °C buffering distance to the suspected drop-off temperature of 67 °C, 65 °C was chosen as the target temperature in subsequent cultivations.

### Investigating growth capabilities on additional carbon sources

With an established defined minimal medium and optimal growth conditions, the carbon source utilization of *G.* sp. LC300 was investigated using a range of monosaccharides, disaccharides, polysaccharides, organic acids, and glycerol (Table 1). Of the tested monomeric sugars, growth was observed on glucose, xylose, and galactose, but not on rhamnose, arabinose, or fructose. In line with these observations, the *G.* sp. LC300 genome lacks predicted genes for both the phosphorylative and non-phosphorylative pathways for rhamnose utilization, as well as arabinose isomerase for arabinose utilization. On the disaccharide sucrose, *G.* sp. LC300 displayed a growth rate close to that on glucose, likely mediated by the predicted sucrose phosphotransferase system (PTS) (IB49_01790). Using this transporter, sucrose would be phosphorylated to sucrose-6-phosphate, likely followed by hydrolysis to fructose and glucose-6-phosphate by the predicted sucrose-6-phosphate hydrolase encoded by IB49_09760. The observed biomass yield on sucrose indicated dissimilation of both the glucose and fructose moieties (data not shown). Together with the absence of accumulation of fructose in the medium, this makes it likely that fructose was subsequently used by the predicted fructokinase (IB49_08420). Together with the abovementioned absence of growth on fructose as the sole carbon source, this suggests that while fructose can be metabolized when already in the cell, *G.* sp. LC300 may lack a functional fructose uptake system.

**Table 1:** Achieved growth rates and yields on different carbon sources

Carbon source	Growth rate (h^−1^)
Glucose	2.2 (± 0.04)
Xylose	1.25 (± 0.01)
Galactose	0.72 (± 0.03)
Rhamnose	No growth
Arabinose	No growth
Fructose	No growth
Lactose	No growth
Maltose	0.76 (± 0.01)
Sucrose	1.91 (± 0.01)
Cellobiose	1.89 (± 0.05)
Starch	1.42 (± 0.06)
Mannitol	1.56 (± 0.04)
Glycerol	1.95 (± 0.002)
Citrate	No growth
Acetate	0.69 (± 0.02)

Values are mean of triplicate shake flask cultivations, ± standard deviation. Glycerol data were collected from duplicate bioreactor cultivations

Growth was further observed on all tested disaccharides except lactose. Since the *G.* sp. LC300 genome lacks an annotated beta-galactosidase, it is likely that the organism is unable to cleave the alpha(1–4) glycosidic bond in lactose to access the monomeric sugar moieties. Of the tested disaccharides and polysaccharides that yielded growth, the growth rate was the lowest on maltose (0.76 h^−1^). Despite this observed growth on maltose, there are no annotated genes connected to maltose import in the *G.* sp. LC300 genome. However, manual alignment of the *Bacillus subtilis* maltose-specific PTS resulted in hits to the predicted glucose-specific PTS of *G.* sp. LC300, and alignment of the *B. subtilis* maltodextrin ABC transporter MdxEFG resulted in hits with E-values lower than 10^–60^ for each gene to a predicted ABC transporter in *G.* sp. LC300 (Supplementary Table 4). We hypothesize that promiscuous activity of either of these systems is responsible for the observed maltose uptake.

Interestingly, despite the observed growth on starch (1.42 h^−1^), there is currently no annotated alpha-amylase in the *G.* sp. LC300 genome. Manual alignment of the *Geobacillus stearothermophilus* alpha-amylase AmyS resulted in a hit (*E* value 10^–56^) to the hypothetical protein IB49_08110, which is a likely candidate for extracellular starch degradation. Like growth on sucrose, when supplied with cellobiose, *G.* sp. LC300 displayed a growth rate (1.89 h^−1^) close to that on glucose. When aligning the cellobiose-specific PTS genes *celABC* from *G. stearothermophilus* (Lai and Ingram 1993) and cellobiose-specific ABC transporter gene *cebE* from *Streptomyces avermitilis* to the *G.* sp. LC300 genome, no hits were found. However, three genes (IB49_08530, IB49_08540, and IB49_14730) encoding putative phospho-beta-glucosidases suggest that *G.* sp. LC300 has the capacity of degrading cellobiose-6-phosphate to glucose-6-phosphate and glucose that can in turn enter the central metabolism.

Glycerol supported growth with a growth rate of 1.95 h^−1^, to our knowledge the highest ever growth rate reported on this substrate. Glycerol is likely imported through the pore-type mechanism of the suggested glycerol uptake facilitator protein (GlpF, IB49_17040), followed by activation through glycerol kinase. Although no glycerol kinase is annotated in the *G.* sp. LC300 genome, alignment of the *B.* subtilis *glpK* resulted in a hit with E-value 0.0 in a region just downstream of *glpF* supporting the existence of this hypothesized degradation pathway.

Of the organic acids tested, growth was only supported on acetate (0.69 h^−1^). Similarly to *E. coli,* (Wolfe 2005) acetate is likely taken up with the assistance of an acetate permease (IB49_14930), followed by conversion to acetyl-CoA by one of two predicted pathways. The AMP-ACS pathway comprises acetyl-CoA synthetase (IB49_05885), converting acetate to acetyl-CoA by hydrolysis of one ATP to AMP and pyrophosphate. The second pathway (PTA-ACKA) relies on acetate kinase (IB49_05785) converting acetate to acetyl phosphate, and acyltransferase (IB49_09645) converting acetyl phosphate to acetyl-CoA.

### Medium finalization and optimal growth characterization

**Fig. 6:**
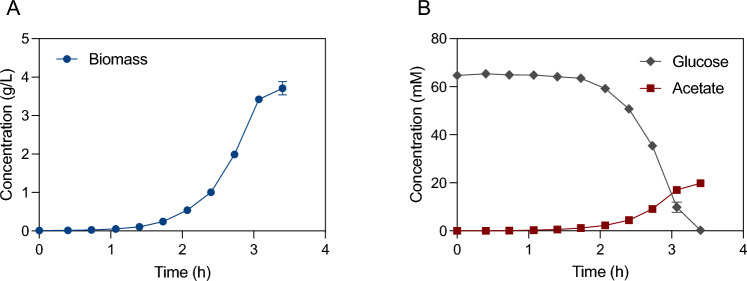
Growth, glucose consumption, and acetate accumulation by *G.* sp. LC300 cultivated at 65 °C and pH 6.75. *Y*-axes represent concentration of biomass (**A**) and metabolites (**B**). *X*-axes represent time after inoculation. Data points represent mean of triplicate bioreactor cultivations with error bars representing standard deviation

With the medium composition and optimal growth conditions established, final bioreactor cultivations were performed to characterize the growth and byproduct formation of *G.* sp. LC300. For this cultivation, one additional modification of the medium was made upon observing a precipitate after 7 days of storing the complete medium at room temperature. While this is not critical for short batch experiments at the high growth rate of *G.* sp. LC300, for longer running chemostat or fed-batch cultures, precipitation in a medium feed vessel could be detrimental. To solve this, 0.3 g L^−1^ of the chelating agent nitrilotriacetic acid (NTA) was added to the medium, combined with a reduction of the magnesium content of 25%. With these changes, no precipitation was seen over several weeks. The final composition, denoted minimal LC300 growth medium (MLGM), used for this experiment can be seen in Table 2. As indicated in earlier experiments, the maximum biomass specific growth rate was 2.20 (± 0.04) h^−1^, until the oxygen transfer rate became limiting at ~ 2 g_DW_ L^−1^. To our knowledge, this is the fastest ever reported growth rate on any carbon source in a defined medium. The biomass yield during this fully aerobic phase was 0.35 g_DW_ g_glc_^−1^ At the point of carbon depletion, the cell mass was ~ 3.5 g L^−1^, resulting in an overall biomass yield on glucose of 0.30 g_DW_ g_glc_^−1^. The glucose was consumed with a maximum biomass specific consumption rate of 35.7 (± 1.1) mmol g_DW_^−1^ h^−1^, and the sole byproduct before oxygen limitation was acetate with a maximum biomass specific production rate of 10.1 (± 0.56) mmol g_DW_^−1^ h^−1^. With these results, the MLGM was confirmed to support growth until carbon depletion using at least 10 g L^−1^ glucose. Furthermore, cultivation settings of 65 °C and pH 6.75 were confirmed to be optimal for the fast growth of *G.* sp. LC300 (Fig. 6).

**Table 2:** Composition of the minimal LC300 growth medium

Component	Concentration
K_2_HPO_4_	9 mM
KH_2_PO_4_	0.7 mM
(NH_4_)_2_SO_4_	32.5 mM
NaCl	13 mM
Nitrilotriacetic acid	1 mM
MOPS (for shake flask cultivations)	50 mM
MgCl_2_	15 mM
CaCl_2_	0.45 mM
Biotin	0.4 μM
Vitamin B12	3.7 nM
FeSO_4_	36 μM
Wolfe’s minerals	10 mL/L

## Conclusion

In this study, we investigated the rapid metabolism of *Geobacillus* sp. LC300. A defined minimal growth medium supporting robust growth for *G.* sp. LC300 was developed, providing a valuable tool for further studies of its physiology. Furthermore, we identified an auxotrophy in *G.* sp. LC300 for vitamin B12 and biotin, revealing a new aspect of its metabolism. This finding not only deepens our understanding of the bacterium's nutritional requirements but also reduces the medium cost by eliminating the supplementation of eight additional vitamins previously used while maintaining the same fast growth. We also investigated the effect of temperature and pH on *G.* sp. LC300 growth using design of experiments. The results showed an optimal growth temperature several degrees lower than previously reported, providing important insights for future efforts to optimize growth conditions for specific industrial applications. Lastly, we found that *G.* sp. LC300 can rapidly utilize a range of biotechnologically interesting saccharides, alcohols, and organic acids as carbon sources, with the fastest ever reported growth rate in defined medium on both glucose and glycerol. This expanded range of substrates provides valuable options for utilizing industrial byproducts and waste streams, which could lead to more sustainable and cost-effective processes. Together, these findings pave the way for further quantitative physiological studies on *G.* sp. LC300, unlocking the secrets of its fast metabolism and advancing its industrial relevance.

### Supplementary Information

Below is the link to the electronic supplementary material.

Supplementary file1 (DOCX 27 KB)

## Data Availability

All experimental data are available upon request.
