# Supplementary material for: Insights into the rapid metabolism of Geobacillus sp. LC300: unraveling metabolic requirements and optimal growth conditions
Source: Extremophiles. 2023 Dec 1;28(1):6. doi: 10.1007/s00792-023-01319-x (PMC10689506; doi:10.1007/s00792-023-01319-x)
Supplement: Supplementary file 1 — Supplementary file1 (DOCX 27 KB) [file 792_2023_1319_MOESM1_ESM.docx]

Supplementary information

Example calculation of elemental requirements for 5 g/L biomass

$$Amount substrate needed=\frac{fraction of element in biomass}{fraction of element in substrate}$$

**Nitrogen:**

Nitrogen source: (NH_4_)_2_SO_4_

M_w_((NH_4_)_2_SO_4_): 132.14 g/mol

Fraction of element in biomass (Stable 2): 14.61%

|  | $\frac{5 g_{x}\times14.61\%}{\frac{14\times2}{132.14}}=3.44 g_{(NH4)2\mathrm{SO}4}$ | (1) |
| --- | --- | --- |

*To ensure carbon limitation at 5 g_x_. an additional 25% of each substrate was added in the medium recipe.*

**Amount biomass supported in Wolfe’s medium**

Nitrogen source: NH_4_Cl

Amount NH_4_Cl in Wolfe’s medium: 0.5 g/L

M_w_(NH_4_Cl): 53.49 g/mol

|  | $\frac{0.5 g_{\mathrm{NH}4\mathrm{Cl}}\times\frac{14}{53.49}}{14.61\%}=0.895 g_{x}$ | (2) |
| --- | --- | --- |

Table 1. Statistical analysis (ANOVA) of design of experiments of temperature and pH

| Growth rate | DF | SS | MS (variance) | F | p | SD |
| --- | --- | --- | --- | --- | --- | --- |
| Total | 11 | 38.1051 | 3.4641 |  |  |  |
| Constant | 1 | 37.1313 | 37.1313 |  |  |  |
|  |  |  |  |  |  |  |
| Total corrected | 10 | 0.973818 | 0.0973818 |  |  | 0.312061 |
| Regression | 4 | 0.937214 | 0.234304 | 38.4059 | **0** | 0.484049 |
| Residual | 6 | 0.0366043 | 0.00610071 |  |  | 0.0781071 |
|  |  |  |  |  |  |  |
| Lack of Fit | 4 | 0.0328043 | 0.00820107 | 4.31636 | **0.197** | 0.0905598 |
| (Model error) |  |  |  |  |  |  |
| Pure error | 2 | 0.0038 | 0.0019 |  |  | 0.043589 |
| (Replicate error) |  |  |  |  |  |  |
|  |  |  |  |  |  |  |
|  | N = 11 | Q2 = | 0.85 | Cond. no. = | 7.46 |  |
|  | DF = 6 | R2 = | 0.962 | RSD = | 0.07811 |  |
|  |  | R2 adj. = | 0.937 |  |  |  |

Table 2. Elemental composition of the *G.* LC300 biomass as predicted by iGEL604

| Element | Mol per mol biomass | Mol per C-mol biomass | Molecular weight | g per c-mol | % in biomass |
| --- | --- | --- | --- | --- | --- |
| C | 36.23 | 1 | 12 | 12.000 | 45.93 |
| H | 65.92 | 1.8195 | 1 | 1.819 | 6.96 |
| N | 9.88 | 0.2727 | 14 | 3.818 | 14.61 |
| O | 16.68 | 0.4604 | 16 | 7.366 | 28.19 |
| P | 1.14 | 0.0315 | 31 | 0.975 | 3.73 |
| S | 0.16 | 0.0044 | 32 | 0.141 | 0.54 |
| Fe | 0.004 | 0.0001 | 55.8 | 0.006 | 0.02 |
|  |  |  | **Sum** | **26.127** |  |

Table 3. Experimental data used for input in the MODDE DoE model.

| **Temperature (°C)** | **pH** | **Growth rate** |
| --- | --- | --- |
| 62 | 6.5 | 2.06 |
| 68 | 6.5 | 1.55 |
| 62 | 7 | 2 |
| 68 | 7 | 1.67 |
| 60 | 6.75 | 2 |
| 70 | 6.75 | 1.34 |
| 65 | 6 | 1.52 |
| 65 | 7.5 | 1.56 |
| 65 | 6.75 | 2.15 |
| 65 | 6.75 | 2.14 |
| 65 | 6.75 | 2.22 |

Table 4. *G.* LC300 genes matched from aligning *Bacillus subtilis* genes

| **Sugar importers** | **Description** | **LC300 locus tag** | **E-value** | **Identities** | **Added to model** |
| --- | --- | --- | --- | --- | --- |
| Maltose ABC transporter | MdxE, periplasmic maltose binding protein from bsu | IB49_13790 | 1.00E-60 | 34% | yes |
| Maltose ABC transporter | MdxF, permease from B sub | IB49_13795 | 2.00E-102 | 48% | yes |
| Maltose ABC transporter | MdxG, permease from B sub | IB49_13800 | 8.00E-76 | 49% | yes |
| Maltose ABC transporter | MsmX, ATP binding subunit from Bsub | IB49_13425 | 0.00E+00 | 76% | yes |
| Glycerol import | GlpF, Pore for glycerol diffusion | IB49_17040 |  |  | yes |
| Glycerol kinase | GlpK, Glycerol kinase from B.sub | not annotated. IB49_17045 suggested | 0.00E+00 | 81% | yes |
